# Head and neck vessel magnetic resonance angiography appearance and artifacts after therapeutic intravenous ferumoxytol infusion

**DOI:** 10.1259/bjrcr.20230014

**Published:** 2023-05-14

**Authors:** James Cody Hanreck, Myroslav Gerasymchuk, Ameya P. Nayate

**Affiliations:** 1 University Hospitals Cleveland, Cleveland, Ohio, United States

## Abstract

Intravenous ferumoxytol infusions are an effective treatment option for iron deficiency anemia. Ferumoxytol contains a superparamagnetic iron oxide core which causes artifacts on multiple MRI brain sequences. However, in our experience, there is not much information on the appearance of intracranial and neck vessels on MR angiography (MRA) after recent therapeutic i.v. administration of ferumoxytol. MRA is an integral part of the work-up for multiple diseases processes including for acute stroke and for detection of aneurysm(s), vasculopathy/vasculitis, vascular malformations, among others and are often performed without the acquisition of MRI brain. Without proper knowledge of the appearance of vessels after administration of i.v. feruomoxytol, radiologists may misinterpret the findings leading to unnecessary further investigation or errant diagnosis. We present the case of a patient who underwent MRI brain and MRA head and neck imaging after recent therapeutic i.v. infusion of ferumoxytol and discuss relevant imaging findings and imaging artifact caused by this medication.

## Clinical presentation

An 85-year-old female presented to the emergency room after a mechanical fall and with slurred speech. Patient subsequently underwent a MRI brain and MR angiography (MRA) head and neck to evaluate for potential neurological causes for her symptoms. Review of the patient’s medical chart revealed that she had received 510 mg i.v. feruomyxtol infusion for iron deficiency anemia approximately 52 h (~2.17 days) prior to undergoing a MRI brain and MRA head and neck.

## Investigations/imaging findings

**Figure 1.**
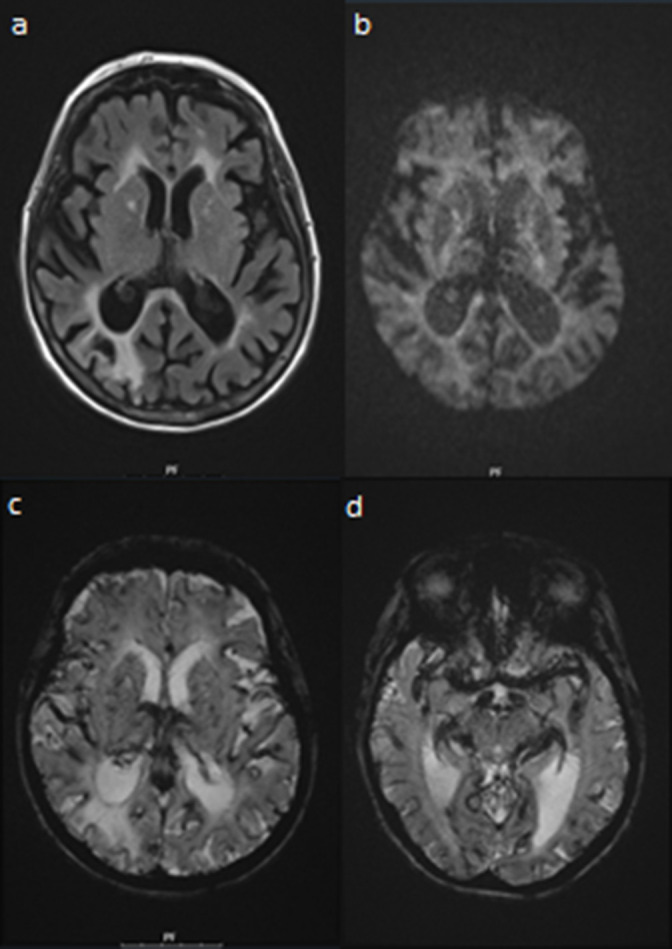
MRI of the brain in a patient 2 days after receiving therapeutic ferumoxytol infusion. (**a**) FLAIR images demonstrate dark signal at the junction between subarachnoid CSF and the surface of the brain due to intravascular ferumoxytol. (**b**) Diffusion-weighted images demonstrate no diffusion restriction abnormality and diminished signal in the subarachnoid space due to intravascular ferumoxtyol. (**c**) Gradient echo imaging demonstrates susceptibility artifact within all visualized intracranial vessels, sparing the parenchyma of the brain due to intravascular ferumoxytol. (**d**) A more inferior gradient echo image demonstrates similar findings. CSF, cerebrospinal fluid; FLAIR, fluid attenuated inversion recovery.

**Figure 2.**
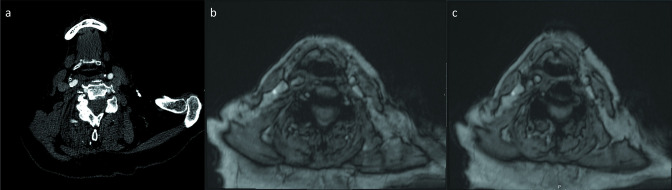
(**a**) CTA of the neck demonstrates atherosclerotic calcification in the right carotid bulb. (**b**) On MRA of the neck, it is more difficult to see these right carotid bulb calcifications. (**c**) Slightly more inferior MRA neck image shows hypointense signal along the surface of the bilateral common carotid arteries due to presence of intravascular ferumoxytol. CTA, CT angiography; MRA, MR angiography.

**Figure 3.**
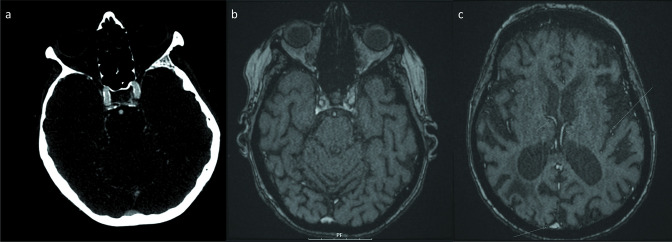
(**a**) CTA of the head demonstrates cavernous right internal carotid artery calcifications. (**b**) On MRA of the head, these cavernous right internal artery calcifications are not seen. (**c**) More superior MRA head image shows hypointense signal along the surface of the distal left middle cerebral artery branches, internal cerebral veins, and the superior sagittal sinus (arrows) due to presence of intravascular feuromoxytol. CTA, CT angiography; MRA, MR angiography.

**Figure 4.**
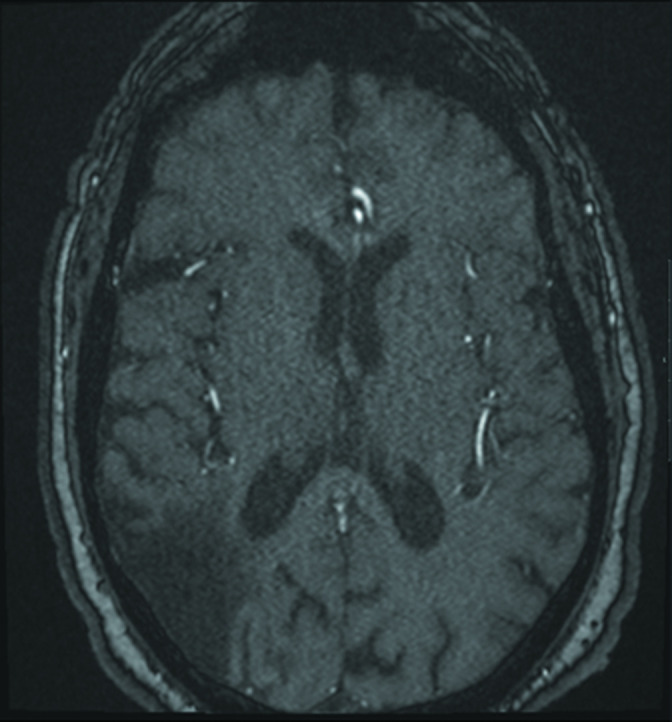
MRA head images from a separate patient obtained from the same scanner with identical parameters demonstrate no susceptibility artifact in the patient’s vessel walls. Note is made of encephalomalacia in the right temporal/occipital region. MRA, MR angiography.

MRI of the brain was performed on a Siemens Healthineers MAGNETOM Aera 1.5 T machine and demonstrated no bright signal abnormality on the diffusion-weighted sequence or FLAIR sequence in the sulci (Figure 1a and b) but demonstrated “blooming” artifact within the vessel walls on the GRE sequence (Figure 1c and d). MRA of the head and neck demonstrated patent arteries and susceptibility artifact along the walls of the arteries, veins, and dural venous sinuses (Figures 2c and 3c). MRA of the head and neck also showed questionable right anterior cerebral artery narrowing and apparent bilateral posterior cerebral artery narrowing with questionable focal occlusion at the left P1/P2 junction (not shown), but no narrowing in the visualized neck arteries. Due to patient motion somewhat obscuring evaluation on MRA, a CT angiography (CTA) of the head and neck was subsequently obtained which showed atherosclerotic calcifications without striking narrowing of the arteries (Figures 2a and 3a). Atherosclerotic calcifications are less apparent on MRA neck (Figure 2b) and MRA head (Figure 3b). MRA head images from a separate patient obtained from the same scanner with identical parameters demonstrate no susceptibility artifact in the patient’s vessel walls, supporting that the susceptibility artifact along the walls of the arteries, veins and dural venous sinuses is secondary to ferumoxytol infusion rather than intrinsic chemical shift artifacts (Figure 4).

## Discussion

Iron deficiency anemia can be treated with i.v. administration of i.v. feuromoxytol.^1^ Two prior studies demonstrated the appearance of the intracranial compartment and abdomen on multiple MRI sequences subsequent to the administration of i.v. feuromoxytol.^2,3^ Our case demonstrates the appearance of the intracranial and neck vessels on MRA after recent therapeutic administration of i.v. feuromoxytol. Feruomoxytol is known to cause the most pronounced artifacts on the gradient echo, susceptibility-weighted imaging, and echoplanar diffusion- weighted sequences since these sequences contain no 180⁰ refocusing pulse to counteract the ferumoxytol-induced T2* shortening affects.^2,3^ Since MRA time of flight is obtained using a conventional 2D or 3D gradient echo sequence and feruomoxytol remains in the intravascular system for 3 days, it is not surprising to see susceptibility artifact from feruomoxytol-induced changes in the walls of the intracranial arteries and veins, potentially related to macrophage uptake.^4^ Although it is important to note flow-related signal is maintained in the lumen of the arteries. Due to dark signal along the walls of the vessels, subtle luminal irregularity or signal from atherosclerotic disease could be masked. In our patient, CTA head and neck study showed no striking narrowing or luminal irregularity of the intracranial or neck arteries but demonstrated regions of atherosclerotic calcifications. Much like on regular MRA studies, atherosclerotic calcifications were not well visualized on MRA after administration of ferumoxytol.

Feuromoxytol is a good MRA contrast agent, particularly in renal failure patients as an alternative to gadolinium-based i.v. contrast.^4^ When it is utilized as such, i.v. feuromoxytol is administered immediately prior to imaging at a significantly lower dose compared to therapeutic dose (1.25–2 mg/kg compared to 510 mg infusion) and the lower dose allows T1 relaxation effects to cause hyperintense signal on *T*_1_ weighted imaging.^4^ However, at higher concentration of i.v. feuromoxytol, reduced T2 and T2* relaxation effects prevail causing markedly reduced signal on the *T*_2_* weighted imaging, like in our case. It is important to note that our case demonstrates dark signal along the walls of the vessels as a delayed manifestation of therapeutic feuromoxytol administration as imaging was obtained approximately 52 h after the patient’s dose was administered.

Given the artifacts feruoxymotol can cause on MRA head and neck imaging, CTA may be a valid and better substitute to evaluate the head and neck arteries in patients who received therapeutic i.v. feruoxymotol in the last few weeks to days. If a patient cannot receive iodinated contrast and MRA is required, the patient intake questionnaire to obtain MR imaging can ask if the patient received therapeutic i.v. feruoxymotol in the last 3 days to weeks so that the radiologist can avoid making an errant diagnosis and to avoid confusion. Importantly, radiologists should be aware that flow-related signal will remain within the vessels even after recent administration of i.v. feruomoxytol, noting that more investigation is needed to further evaluate the dose and duration effects of therapeutic feruomoxytol on MRA imaging.

## Learning points

- I.v. ferumoxytol infusions treat iron deficiency anemia.
- Therapeutic i.v. infused ferumoxytol causes susceptibility artifact along the vessel walls on MRA but flow-related signal within the vessel is not strikingly attenuated compared to CTA.
- For patients who received therapeutic i.v. ferumoxytol in the last few days or weeks, a CTA head and neck study might be a valid and better substitute to evaluate the head and neck arteries. If MRA is required, a patient intake questionnaire to obtain MR imaging can ask if the patient received therapeutic i.v. feruoxymotol in the last 3 days to weeks. This would reduce the probability of misdiagnosis or confusion requiring unnecessary additional or follow-up imaging.
